# Analysis of sarcopenic obesity prevalence and diagnostic agreement according to the 2022 ESPEN and EASO Consensus in hospitalized older adults with severe obesity

**DOI:** 10.3389/fendo.2024.1366229

**Published:** 2024-06-20

**Authors:** Ana Lúcia Danielewicz, Alice Marra, Gabriella Tringali, Roberta De Micheli, Laura Abbruzzese, Paolo Fanari, Franco Codecasa, Stefano Lazzer, Vanessa Amaral Mendonça, Ana Cristina Rodrigues Lacerda, Núbia Carelli Pereira de Avelar, Alessandro Sartorio

**Affiliations:** ^1^ Istituto Auxologico Italiano, Istituto di Ricovero e Cura a Carattere Scientifico (IRCCS), Experimental Laboratory for Auxo-endocrinological Research, Verbania, Italy; ^2^ Department of Health Sciences, Graduate Program in Rehabilitation Sciences, Federal University of Santa Catarina, Araranguá, Santa Catarina, Brazil; ^3^ Istituto Auxologico Italiano, Istituto di Ricovero e Cura a Carattere Scientifico (IRCCS), Division of Eating and Nutrition Disorders, Verbania, Italy; ^4^ Istituto Auxologico Italiano, Istituto di Ricovero e Cura a Carattere Scientifico (IRCCS), Division of Pneumological Rehabilitation, Verbania, Italy; ^5^ Department of Medicine, University of Udine, Udine, Italy; ^6^ School of Sport Science, University of Udine, Udine, Italy; ^7^ Department of Physiotherapy, Federal University of the Jequitinhonha and Mucuri Valleys, Diamantina, Minas Gerais, Brazil

**Keywords:** sarcopenia, obesity, prevalence, older adults, aged

## Abstract

**Background:**

Sarcopenic obesity (SO) is a clinical disorder characterized by increased adiposity and decreased muscle mass and function, commonly observed in older adults. However, most of the studies that investigated SO prevalence rates were not based on current standardized diagnostic methods. Thus, this study aims to estimate the prevalence rates of SO and their level of agreement using different instruments proposed by the European Society for Clinical Nutrition and Metabolism (ESPEN) and the European Association for the Study of Obesity (EASO) Consensus, in a sample of hospitalized older adults with severe obesity.

**Methods:**

A cross-sectional study with 90 older adults (≥ 60 years) with severe obesity (body mass index ≥ 35 kg/m/²) seeking an in-hospital multidisciplinary body weight reduction program. Skeletal muscle function was assessed using the five-repetition Sit-Stand test (5-SSt) and Handgrip Strength (HGS). Body composition was evaluated by high percentages of fat mass (FM), low appendicular lean mass (ALM/W), and skeletal muscle mass (SMM/W), adjusted to body weight. The stage of SO was assessed on the presence of at least one comorbidity and specific cut-offs were adopted for each step. All analyses were performed according to gender and age range.

**Results:**

The prevalence rates of SO in the total sample were 23.3%, 25.5%, 31.1%, and 40.0% considering altered values of 5-SSt+FM+ALM/W, HGS+FM+ALM/W, 5-SSt+FMSSM/W, and HGS+FM+SSM/W, respectively. Higher prevalence rates were observed among female and old elderly subgroups, regardless of the diagnostic combination. There were weak agreements between the muscle function tests (5-SSt *versus* HGS) using both muscle mass indexes in the total sample and all subgroups. Moderate agreements were observed between muscle mass indexes (SMM/W *versus* ALM/W) in the total sample, male and younger older adults (using 5-SSt), and strong agreements for men and younger older adults (using HGS).

**Conclusion:**

The discrepancies observed between the prevalence rates and their levels of agreement reinforce the need for new studies in similar populations aiming for better standardization of SO assessment.

## 1 Introduction

The aging process is associated with several changes in the body’s biological functions, leading to significant alterations in metabolism. These changes include an increase in adipose tissue and a decrease in skeletal muscle mass (1). When combined with genetic and environmental factors, these conditions can lead to chronic illnesses, such as obesity and sarcopenia (2).

In the last decade, obesity prevalence has increased significantly in older adults between 2015 and 2018, with approximately 44% of individuals with obesity in the 65–74 age group and 34% in the ≥ 75 years group (3). Moreover, the prevalence of obesity grade II was around 18.6% for men and 24.4% for women after 65 years, while grade III was 5.7% and 11.6% for older men and women, respectively. On the other hand, sarcopenia can be defined as the presence of low skeletal muscle mass and function (4), and its prevalence varies greatly depending on the method/definition adopted. A worldwide meta-analysis of 151 studies with older individuals with an average age of 68.5 years, showed that the prevalence of sarcopenia ranged from 10% to 27% (5).

Although different, both sarcopenia and obesity can coexist and share pathophysiological aspects and unfavorable outcomes, including multimorbidities (6), frailty (7), and higher risk of mortality (8). In this context, sarcopenic obesity (SO) emerges as a clinical disorder characterized by increased adiposity and decreased skeletal muscle mass and function, which affects 11% of older people worldwide with a substantial increase after 70 years (9). However, this prevalence was based on studies that had not used definitions, diagnostic methods, and cutoff points currently standardized for SO evaluation.

In 2022, the European Society for Clinical Nutrition and Metabolism (ESPEN) and the European Association for the Study of Obesity (EASO) released the first Consensus for the standardization of SO evaluation (10). The Consensus presented sarcopenia and obesity as separate phenotypes, reinforcing that the current isolated definitions should not be automatically applied to define SO. According to this new statement, SO definition should include steps of screening, diagnosis, and staging which can be assessed using different instruments to quantity the fat mass and the skeletal muscle mass (e.g. bioelectrical impedance or dual X-ray absorptiometry), different functional/strength tests (e. g. handgrip strength or sit-to-stand test), as well as different cutoff points for each of these steps (11). Moreover, according to the new criteria, the lean mass must be adjusted by body weight instead of height squared, thus differing from the recommendation of most consensus for isolated sarcopenia (12), to consider differences between normal weight individuals and subjects with obesity.

According to the last meta-analysis on this topic, only four studies applied the current ESPEN/EASO recommendation about the use of skeletal muscle mass adjusted for body weight to define SO in older adults. The combined prevalence varied by 15% compared to that estimated with other studies that used isolated definitions of sarcopenia and obesity (13). Furthermore, to the best of our knowledge, no studies analyzed the level of agreement between the different instruments currently recommended by ESPEN/EASO criteria to identify SO in a sample of older people with severe obesity.

Therefore, there is a clear need for new studies using the standardized definition of SO in different populations, especially with varying degrees of obesity, to consider the differences and similarities between the instruments proposed. This information may be useful for the implementation of SO screening and diagnosis in clinical practice (14). Thus, we aimed to estimate the prevalence rates of SO and their level of agreement using different instruments proposed by the ESPEN/EASO Consensus, in a sample of hospitalized older adults with severe obesity seeking an in-hospital multidisciplinary body weight reduction program.

## 2 Methods

### 2.1 Study design and participants

A cross-sectional study was conducted on Italian older adults, of both sexes, suffering from severe obesity, hospitalized between April 2023 and November 2023 at the Division of Pneumological Rehabilitation and the Division of Rehabilitative Medicine, Istituto Auxologico Italiano, IRCCS, Piancavallo-Verbania, Italy.

All patients were hospitalized for a first diagnostic period (3–4 days) immediately followed by a 3-week multidisciplinary integrated body weight reduction program, entailing an energy-restricted diet in combination with physical rehabilitation, psychological counseling and nutritional education (15). All variables analyzed in the present study were collected in the first three days of hospitalization (i.e. before the beginning of the in-hospital 3-week body weight reduction program).

The inclusion criteria were: age ≥ 60 years and Body Mass Index (BMI) ≥ 35 kg/m^2^ (i.e. grade 2 and 3, according to the World Health Organization) (16). Individuals who had prosthetics, a complete inability to walk, or any severe clinical condition that would prevent getting out of bed and/or engaging in moderate physical efforts independently were excluded.

This study was approved by the Ethical Committee of Istituto Auxologico Italiano, IRCCS, Milan, Italy (protocol number: 2023_03_21_07; research code: 01C313, acronym: PREFISAR) and was conducted following the Declaration of Helsinki. All patients provided written informed consent for their participation in the study.

### 2.2 Sociodemographic and lifestyle characteristics

These data were collected through interviews. Sociodemographic characteristics included gender (female/male), age group (60–69; ≥ 70 years), level of education (elementary, middle, high, graduation), and marital status (single, divorced, married, widowed). Furthermore, lifestyle characteristics included use of alcohol (never, monthly or less, 2/4 times a month and ≥ 4 times a week); smoking (never smoked, smoked and stopped and currently smoking); practice of regular physical activity before hospitalization (yes or no).

### 2.3 Assessment of SO according to the ESPEN/EASO Consensus

**Figure 1:**
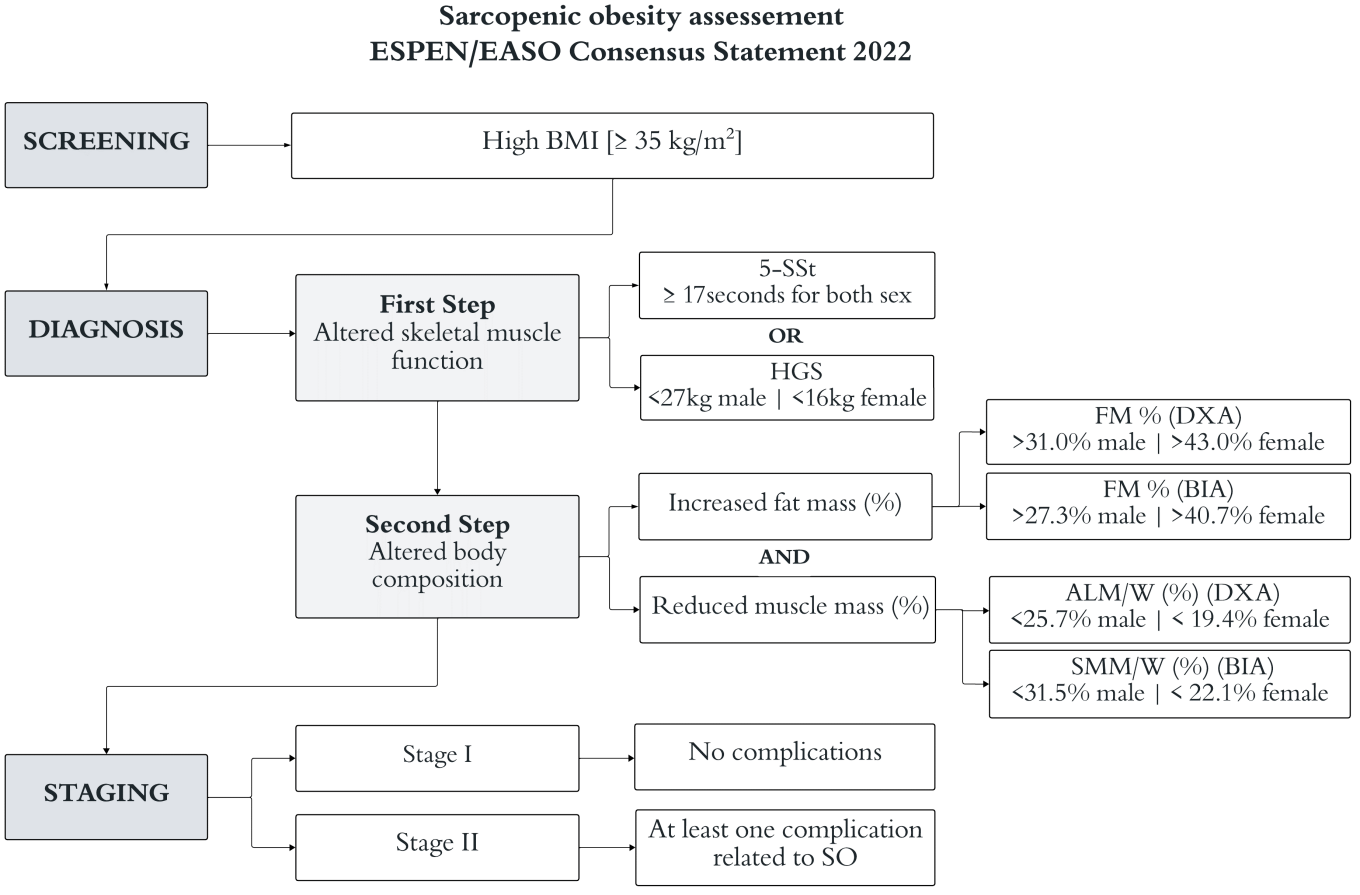
Scheme of steps for the evaluation of sarcopenic obesity (SO) based on the ESPEN/EASO Consensus criteria (10). The cut-offs are described by the SOGLI Expert Panel (11). ALM/W, appendicular lean mass adjusted to body weight; SMM/W, skeletal muscle mass adjusted to body weight; BIA, bioelectrical impedance analysis; BMI, body mass index; DXA, dual X-ray absorptiometry; FM, fat mass; HGS, handgrip strength; 5-SSt, five repetition sit-stand test.

The assessment of SO followed the 2022 ESPEN/EASO Statement Criteria (10). Initially, all participants were screened using high BMI values, after which the altered skeletal muscle function was analyzed using two recommended tests (first step): 1) the five-repetition sit-stand test (5-SSt) and 2) the Handgrip Strength (HGS), followed by assessment of altered body composition (second step) where the diagnosis was confirmed in the presence of both excess of fat mass (FM) and low muscle mass measured by two recommended index: 1) Appendicular lean mass adjusted to body weight (ALM/W) by Dual X-ray Absorptiometry (DXA); 2) Skeletal muscle mass adjusted to body weight (SMM/W) by Bioelectrical Impedance Analysis (BIA). Subsequently, the stage of SO was assessed based on the presence of at least one related comorbidity. Specific cut-offs were adopted for each step according to age group, gender, and examination method following the Sarcopenic Obesity Global Leadership Initiative (SOGLI) Expert Panel recommendations (11). The evaluation scheme is described in **Figure 1**.

#### 2.3.1 Screening variables

The body weight was measured to the nearest 0.1 kg using an electronic scale (Ro WU 150, Wunder Sa.bi., Trezzo sull’Adda, Italy). Standardizing height was determined by a Harpenden Stadiometer (Holtain Limited, Crymych, Dyfed, UK). BMI was calculated by weight (kg) divided by height (m²). These measures were collected by trained professionals, according to the Anthropometric Standardization Reference Manual.

#### 2.3.2 Diagnostic variables

Skeletal muscle function: 1) The HGS was measured with a hand dynamometer (Lafayette Instrument, Inc., Lafayette, United States) in a sitting position, with the shoulder and wrist in a neutral position and the elbow at 90 degrees of flexion (17). Three measurements were performed with the dominant hand, and the average value was used in the analyses. Cut-off values lower than 16 kg for females and 26 kg for males indicated low muscle function (18); 2) The 5-SSt was performed by the participants to measure how fast they could sit and stand five times from a chair with their arms crossed over their chest. The total time in seconds was recorded and a cut-off greater than 17 seconds was adopted for both genders (19).

Two methods were used to evaluate body composition: 1) BIA evaluation was done using a multifrequency tetrapolar impedancemeter (BIA, Human-IM Scan, DS-Medigroup, Milan, Italy) with a delivered current of 800 A at a frequency of 50 kHz. The measurements were taken after 20 minutes of rest in a supine position with relaxed arms and legs without any contact with other body parts (20). The percentage of total FM was used, considering cut-off values greater than 40.7% for females and 27.3 for males (21), and the percentage of SMM/W index considering values lower than 22.1% for females and 31.5% for males (22); 2) DXA evaluation was recorded according to the standardization described by the equipment (Hologic Discovery Wi, Hologic Inc, Waltham, MA, USA) (23). During the evaluation, the participants were positioned in the scanning area of the equipment and a sagittal line passed through the center of all anatomical points of the body. The percentages of total FM provided by the equipment were used for the analysis, considering values greater than 43.0% for females and 31.0% for males (24), and the percentage of ALM/W index based on the total amount of legs and arms lean mass, adopting cut-off values lower than 19.4% for females and 25.7% for males (25).

#### 2.3.3 Staging variables

The following comorbidities were self-assessed to classify the SO stage: back pain, arthritis, cancer, diabetes, hypertension, bronchitis or asthma, sleep apnea, cardiovascular disease, kidney failure, brain stroke, osteoporosis, labyrinthitis, and urinary incontinence. Stage I was considered in the absence of comorbidities and stage II in the presence of at least one comorbidity. Then, all individuals of stage II were divided according to the presence of 1–3 and ≥ 4 of these comorbidities.

### 2.4 Statistical analyses

The description of the sample variables was presented as mean and standard deviation (normal distribution), median and interquartile range (non-normal distribution), and absolute/relative frequencies (categorical variables). The normality of continuous variables was analyzed using the Shapiro-Wilk test and comparisons were made using the Student t-test (normal), and Mann-Whitney test (non-normal) for independent samples. Categorical variables were compared with the Pearson Chi-square test, considering significant p-value < 0.05. All analyses were described according to stratification into subgroups by gender and age range.

The agreement between the prevalence rates was performed using the Cohen Kappa coefficient (k) test. To interpret the agreement analysis, the classification categories proposed by McHugh (26) were considered: 0 to 0.20 represents no agreement; 0.21 to 0.39 represents a minimal agreement; 0.40 to 0.59 represents a weak agreement; 0.60 to 0.79 represents a moderate agreement; 0.80 to 0.90 represents a strong agreement; and above 0.90 represents an almost perfect agreement.

All analyses were performed using the software STATA (version 14.0, Stata Corp., College Station, Texas, EUA) and GraphPad Prism (version 9.3, GraphPad Software, San Diego, CA, USA).

## 3 Results

A sample of 90 patients with severe obesity (median BMI: 43.2 kg/m²; interquartile range: 39.4 - 48.2) with a mean age of 69.4 years (standard deviation ± 5.5 years), hospitalized for a 3-week multidisciplinary body weight reduction program, was admitted to the study. The majority was female (n=47; 52.2%) and with age between 60–69 years (n=48; 53.3%). As far as their sociodemographic characteristics are concerned, significant differences were found only for the marital status variable between genders (p=0.001) and age groups (p=0.001), with a high proportion of married females (38.3%) and older adults ≥ 70 years (40.5%) compared with males (9.3%) and older adults with 60–69 years (10.4%), respectively.

As far as lifestyle is concerned, there was a significant difference in the smoking variable between genders (p=0.001): most females never smoked (57.5%), while most males smoked and stopped (53.5%). Significant differences were also found between genders (p=0.001) for all the variables related to body composition and skeletal muscle function. Females showed a higher percentage ​​of fat mass and lower muscle mass index adjusted for body weight, as well as lower means of HGS ​​and higher 5-SSt values when compared to males. Among different age groups, only HGS values ​​were significantly higher (p=0.033) among the 60–69-year-old group compared to the older group. Description sample details are presented in **Table 1**.

**Table 1:** Sociodemographic, lifestyle, anthropometric, body composition, and skeletal muscle function characteristics in the total sample, according to gender and age group.

Variables	Total (N=90)	Gender			Age group (years)		
		Female (n=47)	Male (n=43)	*p*-value	60–69 (n=48)	≥ 70 (n=42)	*p*-value
Sociodemographic n (%)							
Level of education							
Elementary	26 (28.9)	13 (27.7)	13 (30.2)	0.420	10 (20.9)	16 (38.1)	0.313
Middle	25 (27.8)	16 (34.0)	09 (20.9)		14 (29.1)	11 (26.2)	
High	30 (33.3)	15 (31.9)	15 (34.9)		18 (37.5)	12 (28.6)	
Graduation	06 (10.0)	03 (6.4)	06 (14.0)		06 (12.5)	03 (7.1)	
Marital status							
Single/divorced	20 (22.2)	08 (17.0)	12 (27.9)	0.010*	15 (31.3)	05 (11.9)	0.002*
Married	22 (24.4)	18 (38.3)	04 (9.3)		05 (10.4)	17 (40.5)	
Widowed	48 (53.3)	21 (44.7)	27 (62.8)		28 (58.3)	20 (47.6)	
Lifestyle n (%)							
Smoking							
Never smoked	41(45.6)	27 (57.5)	14 (32.5)	0.050*	19 (39.6)	22 (52.4)	0.464
Smoked and stopped	40 (44.4)	17 (36.1)	23 (53.5)		24 (50.0)	16 (38.1)	
Currently smoking	09 (10.0)	03 (6.4)	06 (14.0)		05 (10.4)	04 (9.5)	
Use of alcohol							
Never	42 (46.7)	26 (55.3)	16 (37.2)	0.240	17 (35.4)	25 (59.5)	0.056
Monthly or less	21(23.3)	10 (21.3)	11 (25.6)		11 (22.9)	10 (23.8)	
≥ 2 times a month	14 (15.6)	07 (14.9)	07 (16.3)		10 (20.8)	04 (9.5)	
≥ 4 times a week	13 (14.4)	04 (8.5)	09 (20.9)		10 (20.8)	03 (7.1)	
Physical activity							
No	75 (83.3)	41(87.2)	34 (79.1)	0.290	40 (83.3)	35 (83.3)	1.000
Yes	15 (16.7)	06 (12.8)	09 (20.9)		08 (16.7)	07 (16.7)	
Anthropometric (median-IQR)							
Height (m)	1.63 (1.54–1.74)	1.54 (1.50–1.60)	1.74 (1.67–1.77)	0.001#	1.65 (1.55–1.76)	1.59 (1.50–1.68)	0.014#
Weight (kg)	114.0 (101.0–131.0)	103.0 (94.2–114.0)	128.0 (116.6–144.7)	0.001#	120.9 (103.5–140.9)	106.2 (97.2–122.5)	0.002#
BMI (kg/m^2^)	43.2 (39.4–48.2)	43.4 (39.4- 47.3)	42.5 (38.3- 48.7)	0.710	44.4 (40.9–48.7)	41.4 (38.7–45.2)	0.041#
Body composition and skeletal muscle function (mean ± SD)							
FM DXA (%)	46.8 (*±* 5.8)	50.1 (± 3.4)	43.2 (± 5.9)	0.001**	47.2 (*±* 6.2)	46.3 (*±* 5.4)	0.479
FM BIA (%)	49.6 (± 7.2)	53.2 (± 6.1)	45.7 (± 6.1)	0.001**	49.5 (*±* 6.7)	49.9 (*±* 7.7)	0.783
ALM/W DXA (%)	20.8 (± 2.6)	19.6 (± 1.8)	22.1 (± 2.7)	0.001**	20.4 (*±* 2.8)	21.2 (*±* 2.3)	0.137
SMM/W BIA (%)	22.7 (± 4.6)	19.3 (± 2.3)	26.3 (± 3.6)	0.001**	22.9 (*±* 3.9)	22.4 (*±* 5.2)	0.570
HGS (kg)	23.0 (± 1.9)	14.6 (± 5.1)	33.1 (± 8.3)	0.001**	25.8 (*±* 11.6)	20.7 (*±* 10.8)	0.033**
5-SSt (s)	16.4 (± 5.6)	17.9 (± 5.9)	15.1 (± 5.0)	0.001**	15.7 (*±* 5.4)	17.4 (*±* 5.7)	0.172

_BMI, body mass index; FM, fat mass; ALM/W, appendicular lean mass adjusted to body weight; BIA, bioelectrical impedance analysis; DXA, dual X-ray absorptiometry; SSM/W, skeletal muscle mass adjusted to body weight; HGS, Handgrip Strength; 5-SSt, five repetitions Sit-Stand test; IQR, interquartile range; SD, standard deviation; *Significant p-value (_<_0.05) for Pearson Chi-square test; #Significant p-value (_<_0.05) for Mann-Whitney test; **Significant p-value (_<_0.05) for Student t-test._

The prevalence rates of SO in total sample varied according to the combination of different diagnostic tests, with values ​​of 23.3%, 25.5%, 31.1%, and 40.0% when considering the combined altered muscle function, fat mass, and muscle mass index assessed by 5-SSt+FM+ALM/W, HGS+FM+ALM/W, 5-SSt+FM+SSM/W, and HGS+FM+SSM/W, respectively. Significant differences were found in the prevalence rates of SO between genders when analyzed by 5-SSt+FM+SSM/W, with 44.7% for females and 16.3% for males (p=0.004), and by HGS+FM+SSM/W with 55.3% for females and 23.2% for males (p=0.002). As far as the age group is concerned, only analysis by HGS+FM+SSM/W showed a significant difference (p=0.007), with 27.1% for the 60–69-year-old group and 54.7% for the ≥ 70-year-old group. All older adults with SO were classified as stage II, with the majority of them reporting ≥ 4 comorbidities, regardless of the diagnostic combination analyzed. More details are presented in **Table 2**.

**Table 2:** Prevalence of sarcopenic obesity (SO) following ESPEN/EASO Consensus in the total sample, according to gender and age groups.

SO diagnosis variables		Gender			Age group		
	Total (N=90)	Female (n=47)	Male (n=43)	*p*-value	60–69 (n=48)	≥ 70 (n=42)	*p*-value
Altered muscle function (first step) (yes, %)							
5-SSt	34 (37.8)	25 (53.2)	09 (20.9)	0.002*	16 (33.3)	18 (42.9)	0.353
HGS	38 (42.2)	28 (59.7)	10 (23.2)	0.001*	14 (29.1)	24 (57.1)	0.007*
SO confirmed (second step) (yes, %)							
5-SSt + FM + ALM/W	21 (23.3)	12 (25.5)	09 (20.9)	0.606	11 (23.0)	10 (23.8)	0.920
HGS + FM + ALM/W	23 (25.5)	13 (27.6)	10 (23.2)	0.632	11 (23.0)	12 (28.6)	0.539
5-SSt + FM + SSM/W	28 (31.1)	21 (44.7)	07 (16.3)	0.004*	13 (27.1)	15 (35.7)	0.378
HGS + FM + SSM/W	36 (40.0)	26 (55.3)	10 (23.2)	0.002*	13 (27.1)	23 (54.7)	0.007*
SO staging (yes, %)							
* Stage I - no comorbidity*	0.0	0.0	0.0	–	0.0	0.0	–
Stage II – at least one comorbidity							
5-SSt + FM + ALM/W							
1–3 comorbidities	03 (13.6)	01 (25.0)	02 (11.1)	0.464	02 (14.3)	01 (12.5)	0.907
≥ 4 comorbidities	18 (26.5)	11 (25.6)	07 (28.0)	0.827	09 (26.5)	09 (26.5)	1.000
HGS + FM + ALM/W							
1–3 comorbidities	05 (22.7)	03 (75.0)	02 (11.1)	0.006*	03 (21.4)	02 (25.0)	0.848
≥ 4 comorbidities	18 (26.5)	10 (23.2)	08 (32.0)	0.431	08 (23.5)	10 (29.4)	0.582
5-SSt + FM + SSM/W							
1–3 comorbidities	02 (9.1)	01 (25.0)	01 (5.6)	0.221	02 (14.3)	0.0 (0.0)	0.262
≥ 4 comorbidities	26 (38.2)	20 (46.1)	06 (24.0)	0.065	11 (32.3)	15 (44.1)	0.318
HGS + FM + SSM/W							
1–3 comorbidities	05 (22.7)	03 (75.0)	02 (11.1)	0.006*	03 (21.4)	02 (25.8)	0.848
≥ 4 comorbidities	31 (45.3)	23 (53.5)	08 (32.0)	0.086	10 (29.4)	21 (61.7)	0.007*

_5-SSt, five repetitions Sit-Stand test; HGS, Handgrip Strength; FM, fat mass; ALM/W, appendicular lean mass adjusted to body weight; SSM/W, skeletal muscle mass adjusted to body weight; *Significant p-value_ <_0.05 for Pearson Chi-square test._

**Figure 2:**
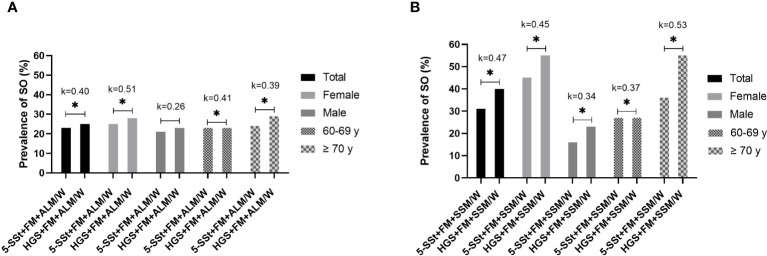
Prevalence of sarcopenic obesity (SO) and level of agreement between muscle function tests (5-SSt versus HGS) using ALM/W index **(A)** and SMM/W index **(B)** in the total sample and according to gender and age group. *Statistically significant difference for Pearson Chi-square test (p-value <0.05). 5SSt, five repetitions Sit-Stand test; HGS, Handgrip Strength; FM, fat mass; ALM/W, appendicular lean mass adjusted to body weight. Agreement analysis: k=0–0.20, no agreement; k=0.21–0.39, minimal agreement; k=0.40–0.59, weak agreement; k=0.60–0.79, moderate agreement; k= 0.80–0.90, strong agreement; k>0.90, almost perfect agreement.

In the analysis of the level of agreement of SO prevalence rates between both muscle function tests (5-SSt *versus* HGS) using ALM/W index to evaluate muscle mass a weak agreement was observed in the total sample (k=0.40; p <0.01), female (k=0.51; p<0.01) and 60–69 years old groups (k=0.41; p<0.01), and a minimal agreement for ≥ 70 years old group (k=0.39; p<0.01) (**Figure 2A**). Similarly, when muscle mass was evaluated with the SSM/W index, weak agreements were detected for the total sample (k=0.40; p<0.01), female (k=0.40; p<0.01) and the ≥ 70 years old groups (k=0.40; p<0.01), and a minimal agreement for male (k=0.40; p<0.01) and 60–69 years old groups (k=0.40; p<0.01) (**Figure 2B**).

**Figure 3:**
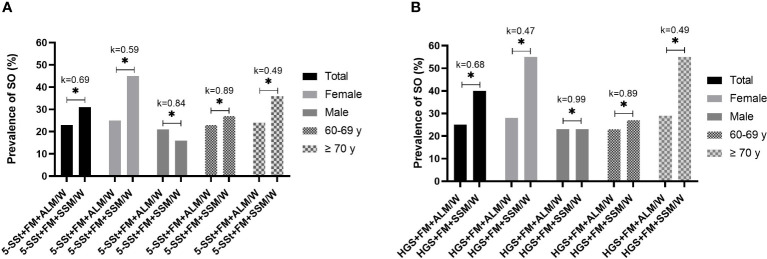
Prevalence of sarcopenic obesity (SO) and level of agreement between muscle mass index (ALM/W *versus* SMM/W) using 5-SSt **(A)** and HGS **(B)** in the total sample and according to gender and age group. *Statistically significant difference for Pearson Chi-square test (p-value <0.05). Abbreviations: 5SSt: five repetitions Sit-Stand test. HGS: Handgrip Strength; FM, fat mass; ALM/W, appendicular lean mass adjusted to body weight. Agreement analysis: k=0–0.20: no agreement; k=0.21–0.39: minimal agreement; k=0.40–0.59: weak agreement; k=0.60–0.79: moderate agreement; k= 0.80–0.90: strong agreement; k>0.90: almost perfect agreement.

In the analysis of the level of agreement of SO prevalence rates between both muscle mass index (ALM/W *versus* SMM/W) considering muscle function evaluated by 5-SSt, moderate agreements were observed in the total sample (k=0.69; p< 0.01), male (k=0.84; p<0.01) and 60–69 years old groups (k=0.89; p<0.01), and a weak agreement for female (k=0.59; p<0.01) and ≥ 70 years old groups (k=0.49; p<0.01) (**Figure 3A**). However, when muscle function was evaluated by HGS, important differences were observed between the subgroups, with a moderate agreement in the total sample (k=0.68; p<0.01), a weak agreement for female (k=0.47; p<0.01) and ≥ 70 years old groups (k=0.49; p<0.01), a strong agreement for 60–69 years old group (k=0.89; p<0.01) and an almost perfect agreement for male group (k=0.99; p<0.01) (**Figure 3B**).

## 4 Discussion

Our study demonstrated that the prevalence rates of SO in the total sample of older adults with severe obesity seeking an in-hospital 3-week body weight reduction program varied depending on the diagnostic combinations taken into consideration. The lowest value observed was 23.0% using the ALM/W index for muscle mass and 5-SSt for muscle function, whereas the highest value was 40.0% using the SMM/W index and HGS for muscle mass and function, respectively. Additionally, significantly higher prevalence rates of SO were observed among females and old elderly group, irrespective of the diagnostic combinations.

According to a recent meta-analysis, the combined prevalence of SO in older adults from four studies utilizing ALM/W or SSM/W was 23.0%, as opposed to only 8.0% when calculated using the ASM/height² index (13). Despite these authors suggested that the normalization to body weight may overestimate sarcopenia in individuals with obesity (27), it is worth noting that obesity results in a lower regenerative capacity of muscle mass. Additionally, even in the absence of an absolute loss of muscle mass, a decrease in muscle mass relative to high total body mass and fat mass may have significant clinical and functional implications (18).

Another recent study showed that the prevalence of SO in Italian older adults with metabolic syndrome assessed using the diagnostic combination SMM/W + HGS was 9.0% (7 out of 61 individuals) (11). However, the cutoff points used to define low muscle mass were higher (37.0% for males and 27.6% for females) compared to those used in our study (31.5% for males and 22.1% for females). It is important to underline that studies using the EASO/ESPEN Consensus to identify the prevalence of SO in older adults are still scarce in the literature and none has ever studied a sample of individuals with severe obesity so far. In another systematic review on the prevalence of SO (5), the studies taken into consideration used definitions specific to the isolated assessment of sarcopenia with different indexes and cutoff points, thus making difficult reliable comparisons.

As observed in our findings, the rates of sarcopenic obesity (SO) were higher in female and old elderly subgroups. In a recent study with Japanese older adults (mean age: 76.5 years), the authors identified 87 individuals with SO, the large majority (64.0%) being females (28). In line with this finding Muollo et al. (29) found that older females with obesity had lower muscle mass than age-matched males with obesity in the upper and lower limbs (- 41% and - 32%, respectively). In the study by Samuel et al. (30), the mean values of both grip and quadriceps muscle strength of older females (mean age 72.4 years) were 56% lower than those of their male counterparts (mean age 71.2 years). Physiological mechanisms such as age-related hormonal levels (growth hormone, insulin-like growth factor 1, sex hormones, etc.), insulin resistance, inflammation, and oxidative stress levels contribute to these more pronounced differences in females and old elderly subgroups. These mechanisms can occur more prominently in the presence of obesity, leading to the formation of a detrimental cycle of degeneration between adipose and muscle tissues (31).

In our study, all patients with SO were classified in stage II (i.e. presence of at least one comorbidity), and most of them reported four or more comorbidities. According to the results presented in the systematic review by Liu et al. (13), in 46 out of 51 analyzed studies, there was an association of SO with comorbidities or adverse events, including an increased risk of stroke, heart and metabolic diseases, decreased physical function, pulmonary and orthopedic diseases. Furthermore, 5 out of 9 studies quantitatively analyzed by the authors showed that older adults with SO displayed a higher prevalence of comorbidities than those with only sarcopenia or only obesity. These findings reinforce the hypothesis that individuals with SO exhibit a more adverse pro-inflammatory and metabolic status, contributing to an increased occurrence of comorbidities (32).

In our study, weak agreements between the prevalence rates of SO in the total sample were found, as well as in subgroups subdivided by gender and age, when comparing the two skeletal muscle function tests (5-SSt versus HGS), regardless of the analyzed muscle mass index. In a study conducted by Muollo et al. (29), a moderate correlation between HGS values and lower limb muscle strength by isokinetic evaluation was observed in older males with obesity and a weak correlation in older females with obesity. On the other hand, a moderate and inverse correlation was found between HGS and 5-SSt for males, whereas no correlation was observed for females. Nevertheless, the comparison of isokinetic assessment with our results should be done with caution, although it has been suggested as a possible instrument for the diagnosis of SO by the ESPEN/EASO consensus. Although the isokinetic assessment showed a positive high correlation with the 5-SSt (33), it may reflect different results in terms of muscle function according to the sex and age of the sample. In another study, similarly to our findings, no concordance was reported between the two screening tests (5-SSt and HGS) for detecting probable sarcopenia in non-obese Brazilian older females (34). The authors suggested that this could be because HGS is a more specific test for assessing strength in the upper limbs, while the 5-SSt evaluates the strength of lower limbs and is commonly used as a proxy measure for physical performance.

As mentioned above, the decline in muscle strength during aging is more evidentin the lower limbs compared to the upper limbs. This finding is attributed to the minor engagement of older adults in physical activities such as walking, running, and stair-climbing, which would naturally have a more significant impact on the lower body (35). Although in the present study, weak correlations between the two muscle function tests were found, these tests still retain their relevance for SO diagnosis, in particular when they are used for assessments of older adults with severe obesity.

Our results showed moderate agreements between the muscle mass indexes (SMM/W versus ALM/W) in the total sample, regardless of the muscle function tests analyzed. Moderate agreement was observed for males and younger older adults (using 5-SSt), as well as strong agreement for males and almost perfect agreement for younger older adults (using HGS). In a recently published study by Juby et al. (36), the BIA equipment with four sensors had high specificity and poor sensitivity for detecting muscle mass in older adults with obesity, while another whole-body BIA with eight sensors had lower specificity and higher sensitivity for the same measures. However, regardless of the BIA equipment used, these authors reported that derived muscle mass showed acceptable comparisons with values obtained by DXA.

It is worth noting that Vieira et al. (37) found moderate agreement between the prevalence rates of SO between BIA and DXA (k=0.43) using the ESPEN/EASO consensus definitions. However, this study focused on an adult sample of individuals after bariatric surgery and did not consider differences between the two muscle function tests. Although moderate correlations were found, it is important to emphasize that both indexes, SMM/W and ALM/W, derived from BIA and DXA, respectively, have divergent points that should be carefully considered when they are used. Furthermore, our results indicated that the HGS appears to be more reliable than the 5-SST in detecting SO among males and younger older adults due to the strong agreements observed when it was used in the muscle mass indexes analysis. This finding aligns with the fact that these subgroups exhibit a greater amount of muscle mass in the upper limbs compared to the lower limbs.

Thus, we believe that our results represent a first step for future research aiming for better standardization of methods for assessing SO, as well as a useful guide for professionals working on hospitalized older adults with severe obesity. Nevertheless, our study has some limitations to be highlighted. Since we used a relatively small sample size of Italian older adults with severe obesity seeking an in-hospital multidisciplinary body weight reduction program, the results cannot be generalized to other populations of older adults. There is still a lack of concrete evidence regarding the best cutoff points of the instruments used for SO diagnosis in older adults with obesity, especially for the SMM/W and ALM/W indexes, which are more recently recommended. Aligning with SOGLI Expert Panel recommendations (11), we chose the best options of values that closely matched our sample of subjects with obesity in terms of ethnicity and age group. However, these cutoff points may not reflect the real changes in muscle mass in severe obese people and may have underestimated the prevalence rates of SO in men and/or overestimated in women.

To conclude, our study showed for the first time that the prevalence rates of SO in hospitalized older adults with severe obesity varied greatly depending on the diagnostic combinations analyzed, according to the recommendations of the ESPEN/EASO Consensus. The lower values were found using ALM/W for assessing altered muscle mass and 5-SSt for muscle function, while higher values were observed using SMM/W for muscle mass and HGS for muscle function. The higher prevalence rates of SO were observed among females and old older adults, regardless of the diagnostic combinations. Additionally, our results showed weak agreement in the prevalence rates of SO for the total sample and subgroups when comparing the two muscle function tests (5-SSt versus HGS), whereas moderate agreements in the total sample, and strong agreements for men and younger older adults were detected in the comparison between the muscle mass indexes (SMM/W versus ALM/W).

Taking into consideration all the results, our study highlights the need for new studies with larger samples and similar populations aiming for better standardization of each of the three stages of SO assessment (screening, diagnosis, and staging) proposed by ESPEN/EASO Consensus. Additionally, further research is required to investigate deeper into the physiopathology of SO, aiming to comprehend the differences in muscle biomarkers that may assist in more accurate diagnosis.

## Data availability statement

The raw data supporting the conclusions of this article will be made available by the authors, without undue reservation.

## Ethics statement

The studies involving humans were approved by Ethical Committee of Istituto Auxologico Italiano, IRCCS, Milan Italy (protocol number: 2023_03_21_07; research code: 01C313, acronym: PREFISAR). Written informed consent was signed by all participants. The studies were conducted in accordance with the local legislation and institutional requirements. The participants provided their written informed consent to participate in this study.

## Author contributions

AD: Conceptualization, Data curation, Formal analysis, Investigation, Methodology, Software, Validation, Visualization, Writing – original draft, Writing – review & editing. AM: Conceptualization, Data curation, Methodology, Validation, Visualization, Writing – review & editing. GT: Data curation, Methodology, Validation, Visualization, Writing – review & editing. RM: Data curation, Methodology, Validation, Visualization, Writing – review & editing. LA: Data curation, Supervision, Validation, Visualization, Writing – review & editing. PF: Data curation, Supervision, Validation, Visualization, Writing – review & editing. FC: Data curation, Supervision, Validation, Visualization, Writing – review & editing. SL: Supervision, Validation, Visualization, Writing – review & editing. VM: Supervision, Validation, Visualization, Writing – review & editing. AL: Supervision, Validation, Visualization, Writing – review & editing. NP: Methodology, Supervision, Validation, Visualization, Writing – review & editing. AS: Conceptualization, Funding acquisition, Methodology, Project administration, Resources, Supervision, Validation, Visualization, Writing – original draft, Writing – review & editing.
